# Effects of altered glycolysis levels on CD8^+^ T cell activation and function

**DOI:** 10.1038/s41419-023-05937-3

**Published:** 2023-07-08

**Authors:** Jiaying Cao, Shan Liao, Feng Zeng, Qianjin Liao, Gengqiu Luo, Yanhong Zhou

**Affiliations:** 1 NHC Key Laboratory of Carcinogenesis, Hunan Cancer Hospital and The Affiliated Cancer Hospital of Xiangya School of Medicine, Central South University, Changsha, Hunan 410013 China; 2 Cancer Research Institute, Basic School of Medicine, Central South University, Changsha, Hunan 410078 China; 3 Hunan Key Laboratory of Cancer Metabolism, Hunan Cancer Hospital and the Affiliated Cancer Hospital of Xiangya School of Medicine, Central South University, Changsha, Hunan 410013 China; 4 Department of Pathology, The Third Xiangya Hospital, Central South University, Changsha, Hunan 410013 China; 5 Department of Pathology, Xiangya Hospital, Basic School of Medicine, Central South University, Changsha, Hunan 410008 China

**Keywords:** Cell death and immune response, Cell growth

## Abstract

CD8^+^ T cells are an important component of the body’s adaptive immune response. During viral or intracellular bacterial infections, CD8^+^ T cells are rapidly activated and differentiated to exert their immune function by producing cytokines. Alterations in the glycolysis of CD8^+^ T cells have an important effect on their activation and function, while glycolysis is important for CD8^+^ T cell functional failure and recovery. This paper summarizes the importance of CD8^+^ T cell glycolysis in the immune system. We discuss the link between glycolysis and CD8^+^ T cell activation, differentiation, and proliferation, and the effect of altered glycolysis on CD8^+^ T cell function. In addition, potential molecular targets to enhance and restore the immune function of CD8^+^ T cells by affecting glycolysis and the link between glycolysis and CD8^+^ T cell senescence are summarized. This review provides new insights into the relationship between glycolysis and CD8^+^ T cell function, and proposes novel strategies for immunotherapy by targeting glycolysis.

## Facts

1. Activation of CD8^+^ T cells by TCR induces a shift in the level of cellular metabolism toward glycolysis, while the synergistic stimulatory effect of CD28 will cause an upregulation of CD8^+^ T cell glycolysis levels to further support their subsequent proliferation and differentiation.
2. The induction of high glycolytic activity in CD8^+^ T cells favors CD8^+^ T cell differentiation toward effector cells, but severely impairs the survival of long-lived memory cells.
3. For effector CD8^+^ T cells, changes in their glycolysis play an important role in the production of IFN-γ, whereas downregulation of glycolysis levels is detrimental to their production of relevant cytokines and immune functions. Therefore, it is crucial to find ways to target glycolysis to restore CD8^+^ T cell effector functions.
4. Metabolic dysregulation caused by changes in glycolytic activity affects not only the function of CD8^+^ T cells but also their effector functions, and selective enhancement of glycolysis will further restore these functions.
5. Glycolysis might affect the partial failure of CD8^+^ T cells through the mTOR pathway and adversely affect the production of IFN-γ. In addition, the balance between glycolysis and FAO is associated with the long-term survival of memory CD8^+^ T cells.

## Introduction

CD8^+^ T cells, also known as cytotoxic T lymphocytes (CTLs), interact with the MHCI complex, and are one of the most important components of the body’s adaptive immune system, playing an important role in the adaptive immune response to intracellular pathogens and cancers [1, 2]. Under the coordinated activation of three signals, including T cell receptor (TCR), costimulation, and inflammatory cytokines, naive CD8^+^ T cells undergo clonal expansion and proliferation [3], followed by differentiation into effector CD8^+^ T cells [3–6]. Effector CD8^+^ T cells induce cell death by secreting cytotoxic proteins (including perforin and granzymes), which selectively trigger the activation of Caspase, leading to cell apoptosis [7–9]. In addition, effector CD8^+^T cells also release cytokines to exert their immune function, such as tumor necrosis factor (TNF) -α, which indirectly kills target cells, and interferon (IFN) -γ, which can inhibit virus replication and enhance specific antigen presentation [10, 11]. However, excessive cytokine may also lead to immune overactivation and threaten life [12, 13]. Subsequently, most effector CD8^+^ T cells undergo shrinkage and then apoptosis. However, a small number of them survive and are transformed into memory CD8^+^ T cells, which can be rapidly activated and undergo an immune response when they encounter antigens again [14], thereby effectively removing viruses or tumor cells. Thus, memory CD8^+^ T cells play a role in the body’s anti-tumor and anti-infection immune processes, exerting a crucial function in the durable protection against intracellular pathogens and tumors [15]. Unlike acute microbial infection, under the action of multiple mechanisms of the tumor microenvironment (TME), tumor cells usually persist and undergo immune escape to avoid the recognition attacks by immune cells such as T cells [16]. In this process, tumor cells are protected from attack by downregulating MHC and increasing immune checkpoint ligands (e.g., programmed death-ligand 1/2, T cell immunoglobulin and ITIM domain (TIGIT) and T cell immunoglobulin and mucin domain 3 (TIM-3)) as well as transforming growth factor (TGF)-β and interleukin (IL) expression, which often causes T cell depletion [17–19]. Therefore, intervening in the adaptive immune response by targeting CD8^+^ T cell checkpoints and depleted CD8^+^ T cells in the face of this type of immune escape is beneficial for tumor elimination. Currently, combining immune checkpoint blockade (ICB) and other anticancer treatments have achieved significant success in various cancers [20–22].

In general, naive CD8^+^ T cells rely mainly on ATP produced by mitochondrial oxidative phosphorylation (OXPHOS) for survival [23]. Meanwhile, to prevent atrophy of quiescent T cells, IL-7 signaling mediates the activation of protein kinase B (AKT) through signal transducer and activator of transcription 5 (STAT5), to regulate glucose transporter (GLUT) 1 expression to promote glucose uptake [24], which in turn serves as fuel for OXPHOS. Naive CD8^+^ T cells are further activated and differentiated into effector CD8^+^ T cells, which rely mainly on glycolysis, fatty acid synthesis, and amino acid metabolism to promote cell proliferation and cytokine secretion. By contrast, memory CD8^+^ T cells rely on the tricarboxylic acid cycle and fatty acid oxidation to maintain their lifespan [25–27]. Glycolysis is a metabolic pathway of cells, glucose generates pyruvate and ATP under the action of key enzymes of glycolysis, including GLUT, hexokinase (HK), phosphofructosekinase (PFK) and pyruvate kinase (PKM) [28]. Under the sufficient oxygen condition, pyruvate is oxidized by pyruvate dehydrogenase to produce acetyl coenzyme A, which enters the tricarboxylic acid (TCA) cycle and OXPHOS; whereas when oxygen is absen, pyruvate is catalyzed by lactate dehydrogenase to produce lactate [29]. Increasingly, it has been shown that in addition to the significant effects of changes in kinase activity on glycolytic activity and cell growth [30], signaling pathways such as PI3K/AKT/mTOR also play an important role in cell survival and development by mediating glycolysis-related processes [31]. An increasing number of studies have shown that changes in CD8^+^ T cells’ glycolysis and OXPHOS have different regulatory effects on CD8^+^ T cell activation, differentiation, and function.

This paper summarizes the relationship between altered levels of CD8^+^ T cell glycolysis and their activation, proliferation, and differentiation. It suggests that the upregulation of glycolysis levels after CD8^+^ T cell activation provides metabolic support for their further proliferation and differentiation. At the same time, glycolysis is also crucial for the effector functions of CD8^+^ T cells and the reactivation and effector functions of memory CD8^+^ T cells. In addition, this paper discusses the potential link between CD8^+^ T cell glycolysis and their functional failure and recovery, providing a new direction for targeting CD8^+^ T cell glycolysis to improve adaptive immune function.

## Association between glycolysis levels and CD8^+^ T cell activation, proliferation, and differentiation

### Changes in the glycolysis levels of CD8^+^ T cells after activation

The activation of naive CD8^+^ T cells is stimulated by signals from the TCR, which affect the cells’ ability to remain quiescent or to be activated, and to produce cytokines, which induce cell proliferation and differentiation [32]. TCR can rapidly induce glycolysis in the early stages of CD8^+^ T cell activation, which is independent of de novo transcription and translation, while acting independently of the joint action of CD28 and AKT. Moreover, this process does not involve an increase in glucose uptake or glycolytic enzyme activity during glycolysis. Instead, TCR signaling activates pyruvate dehydrogenase kinase 1 (PDHK1) to inhibit the entry of pyruvate into mitochondria and catalyzes its conversion to acetyl coenzyme A (CoA) for the TCA cycle and its catabolism to lactate via lactate dehydrogenase (LDH) in the cytoplasm. This activation process involves tyrosine kinases lymphocyte cell-specific protein-tyrosine kinase (LCK) and zeta chain of TCR associated protein kinase 70 (ZAP70) binding to and phosphorylating PDHK1, which affects PDH activity [33]. Therefore, we suggest that PDHK1 is an important signal in the early stages of T cell activation and contributes to the induction of a rapid switch in the metabolic pattern of CD8^+^ T cells. While TCR activates CD8^+^ T cells, CD28 promotes their maximal glucose uptake after TCR stimulation by upregulating the expression of GLUT1 on the cell surface [34]. CD28 induces glucose uptake and glycolysis levels to maintain cellular ATP/ADP levels, or the energy required for macromolecular synthesis, by mediating phosphatidylinositol-4,5-bisphosphate 3-kinase (PI3K) and AKT activity [35]. Furthermore, it was found that PQDN (10-dodecyl-6-nitro-pyrimido[4,5-*b*]quinoline-2,4 (*3H,10H*)-dione), a small molecule involved in TCR activation of CD8^+^ T cells, activates the electron transport chain (ETC) in mitochondria by acting on flavin mononucleotide (FMN) to promote the conversion of NADH to NAD^+^, further accelerating mechanistic target of rapamycin kinase (mTOR)/AKT signaling to induce glucose uptake, promote glycolytic pathways and mitochondrial respiration, and activate CD8^+^ T cells [36]. This provides tumor microenvironment (TME)- TCR stimulation deficient conditions by targeting activation-related small molecules to modulate T cell metabolism, thereby promoting their activation. Furthermore, T cell activation is an energy demanding process, and as glucose consumption increases, the insulin receptor (INSR) is upregulated accordingly. In addition, silencing of *Insr* in rats attenuated the cytotoxicity of CD8^+^ T cells toward alloantigens [37]. However, the artificial administration of glucocorticoids (GCs) during the TCR activation phase induced long-term inhibition of glycolysis, which persisted even when the addition of GCs was stopped at a later stage, which significantly inhibited CD8^+^ T cell effector functions and was detrimental to their antitumor activity and further conversion to memory CD8^+^ T cells [38].

**Fig. 1:**
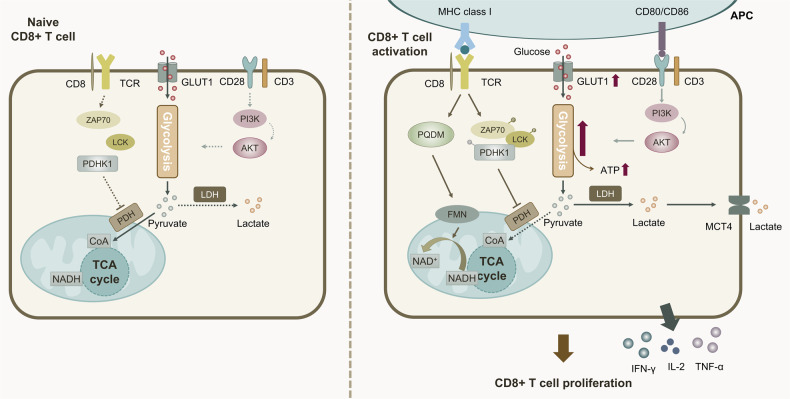
Changes in the level of auto-glycolysis after CD8^+^ T cell activation. After the activation of CD8^+^ T cell surface TCR by MHC class I presentation of antigen presenting cells, Lck and ZAP70 bind to PDHK1 and induce its phosphorylation, which inhibits pyruvate from entering mitochondria and converting to CoA to participate in the TCA cycle. Meanwhile, while pyruvate in the cytoplasm is catabolized to lactate by LDH, causing a shift in the level of cellular metabolism toward glycolysis. Meanwhile, CD80/CD86 presentation activates CD28, which in turn upregulates the expression level of GLUT1 to promote its maximum glucose uptake after TCR stimulation and mediates PI3K and AKT-induced glycolysis levels to maintain cellular ATP/ADP levels or the energy required for macromolecular synthesis.

The above studies suggest that activation of CD8^+^ T cells by TCR induces a shift in the level of cellular metabolism toward glycolysis, while the synergistic stimulatory effect of CD28 will cause an upregulation of CD8^+^ T cell glycolysis levels to further support their subsequent proliferation and differentiation. Increasing numbers of studies have focused on the study of small molecules related to TCR activation, which will provide new ideas for the development of targeted drugs to address the environment of TCR underactivation in the TME by modulating the activation of CD8^+^ T cells and their glycolysis levels (Fig. 1).

### Effect of altered levels of glycolysis on CD8^+^ T cell proliferation

The upregulation of glycolysis levels caused by the activation of naive CD8^+^ T cells correlates positively with their proliferative capacity and also reduces the sensitivity of cell growth to metabolic inhibition, further providing energy and biosynthetic material for their growth, proliferation, and differentiation. Unlike CD4^+^ T cells, the activation of CD8^+^ T cells does not completely lead to a complete shift in their metabolism towards aerobic glycolysis, which still has a requirement for intact OXPHOS [39]. And this specific change in metabolic programming promotes the growth and proliferation of CD8^+^ T cells, while also improving their survival under different nutritional conditions [40]. In this process, phosphatidylinositol-4,5-bisphosphate 3-kinase catalytic subunit delta (p110δ) signaling regulates OXPHOS and aerobic glycolytic processes activated by TCR through activation of AKT, affecting CD8^+^ T cell activation, proliferation, and effector cytokine production [41]. In addition, in gastric cancer, S100 calcium binding proteins S100A8/A9 inhibit CD8^+^ T cell glycolysis through the Toll like receptor 4 (TLR4)/AKT/mTOR pathway, affecting their proliferation and eventually leading to failure [42]. Furthermore, activation of mouse CD8^+^ T cells by CD3 and CD28 shifted their metabolism toward aerobic glycolysis, which would favor further CD8^+^ T cell proliferation [43]. Compared with their wild-type controls, in vitro activation of 2B4 (also known as signaling lymphocytic activation molecule 4 (SLAMF4) or CD244)-deficient (2B4KO) CD8^+^ T cells exhibited higher glycolytic activity and an upregulated gene expression profile [44]. Moreover, CD8^+^ T cells gained a greater proliferative advantage; however, in a glucose-deficient environment, 2B4KO CD8^+^ T cells lost this proliferative advantage, suggesting that 2B4 plays an important role in altering CD8^+^ T cell glucose metabolism to limit their proliferation [44]. Bromodomain protein 4 (BRD4) supports the survival of naïve CD8^+^ T cells by promoting glucose uptake and mitochondrial energy production through the upregulation of MYC and GLUT1 protein levels, whereas the decreased glycolysis activity caused by insufficient glucose uptake in the absence of BRD4 would be detrimental to CD8^+^ T cell proliferation [45]. In contrast, in an investigation of patients with hypopharyngeal cancer, radiotherapy (RT) was found to regulate programmed cell death ligand 1 (PD-L1) by upregulating GLUT1 expression, thereby promoting CD8^+^ T cell proliferation and regulating immune competence [46]. Therefore, PD-L1 might be a potential downstream molecule of GLUT1-mediated glycolysis that affects CD8^+^ T proliferation. Rapid T cell proliferation facilitates an effective adaptive immune response together with subsequent cell death, and studies have shown that induction of glycolysis in CD8^+^ T cells synergizes with complex I and methylation-controlled J protein (MCJ), an inhibitor of OXPHOS, to promote caspase-3 activity, which helps to prevent the accumulation of highly proliferative CD8^+^ T cells and promotes their timely death [47].

Therefore, upregulated glycolysis provides energy for CD8^+^ T cells to adapt to growth and further proliferation. It also suggests the feasibility of promoting the proliferation of CD8^+^ T cells by altering their metabolism to further enhance their immune function. At the same time, appropriate regulation of CD8^+^ T cell glycolysis levels could also help to promote timely death and prevent excessive proliferation, providing new ideas for immunotherapy. However, glycolysis changes have functions in different stages of CD8^+^ T cell activation, and the specific mechanism of action between glycolysis changes and CD8^+^ T cell proliferation has been poorly investigated, which is not conducive to directly affecting the proliferation of CD8^+^ T cells by targeting the key molecules involved. In addition, CD8^+^ T cells proliferation is not completely dependent on glycolysis, and high level of OXPHOS also plays an important role. Unfortunately, the potential relationship between glycolysis and OXPHOS of CD8^+^ T cells is still unclear, with specific mechanisms still to be refined.

### Effect of altered glycolysis levels on CD8^+^ T cell differentiation

During viral infection, T cells adapt to the activated and differentiated state via metabolic reprogramming [48]. Moreover, metabolic alterations induced by external stimuli also have an important impact on CD8^+^ T cell differentiation, because the bioenergetic metabolism of naive CD8^+^ T cells shifts from OXPHOS to aerobic glycolysis and further proliferation in response to antigenic stimulation. Quantification of glucose uptake by CD8^+^ T cells using the fluorescent glucose analogue 2-NBDG (2-(N-(7-Nitrobenz-2-oxa-1,3-diazol-4-yl)Amino)-2-Deoxyglucose) revealed that CD8^+^ T cells that take up large amounts of glucose tend to have a molecular signature of short-lived effectors, suggesting a possible role of glycolysis in the existence of CD8^+^ T cell differentiation toward effector CD8^+^ T cells. Furthermore, overexpression of the glycolysis enzyme phosphoglycerate mutase-1 (PGAM1) severely impaired the ability of CD8^+^ T cells to differentiate into long-lived memory cells after shifting cellular metabolism toward glycolysis [49]. Meanwhile, glycolysis and glutaminolysis are essential for TLR2-mediated T cell activation. IRF4, an important transcription factor in the interferon regulatory factor (IRF) family, plays a crucial role in the immune response by regulating CD8^+^ T cell differentiation [50], and IRF4 and its co-binding partner B cell-activated transcription factor (BATF) are necessary for sustained CD8^+^ T cell effector function [51], while IRF4 regulates the sensitivity of CD8^+^ T cell to IL-2. And TLR2 signaling further upregulates effector CD8^+^ T cell immunoregulatory and functionally relevant gene expression, such as those encoding T-bet and IFN-γ, by increasing the expression of bioenergy metabolism-related genes such as *IRF4* in CD8^+^ T cells and improving their glycolysis and glutaminolysis [52]. Meanwhile, TLR7 regulates the downstream transcription factor *IRF4* via the AKT-mTOR pathway, thereby stimulating metabolic changes in CD8^+^ T cells, including upregulation of glucose uptake and glycolysis, which promotes the effector functions of CD8^+^ T cells in vitro. In contrast, the corresponding deprivation of the mTOR pathway and *IRF4* expression impaired the activation and function of effector CD8^+^ T cells [53]. Although in some cell systems mTOR links PI3K and AKT to the control of glucose uptake and glycolysis, mTORC1 independently controls GLUTs, multiple rate-limiting glycolysis enzymes, cytolytic effector molecules, and a diverse transcriptional program that regulates the expression of essential chemokines and adhesion receptors for T cell migration [54]. TSC complex subunit 1 (TSC1) deletion promoted mTORC1 activity, causing dysregulation of IL-15-stimulated glycolysis and oxidative metabolism, resulting in transient effector cell differentiation, but not memory precursor effector cell generation [55].

However, efficient glycolysis is not conducive to the long-term survival of memory type cells, i.e., the aforementioned metabolic imbalance drives their differentiation into short-lived effectors but impairs the establishment of their immune memory function [49]. Studies have shown that naive CD8^+^ T cells in neonatal cord blood, when activated alone or in the presence of TGF-β or inflammatory cytokines, tend to differentiate into non-classical TC2 cells, accompanied by IL-4 activation, which is associated with reduced expression of glycolysis and increased fatty acid metabolism [56]. The use of the HK2 inhibitor 2-deoxyglucose (2-DG) to limit glycolysis in CD8^+^ T cells facilitates the formation of memory CD8^+^ T cells and enhances their ability to trigger the destruction of established tumors [49, 57]. The formation of CD8^+^ memory T cells was detected in vitro using IL-15 induced by the high expression of phosphoenolpyruvate carboxykinase (PCK1), a key rate-limiting enzyme for gluconeogenesis. PCK1 activity causes highly active gluconeogenesis, catalyzing the generation of glucose 6-phosphate, which enters the pentose phosphate pathway and generates reduced NADPH to ensure high levels of reduced glutathione, while scavenging intracellular free radicals, thus maintaining the long-term survival of memory T cells. The corresponding abolition of PCK1-glycogen-PPP results in a decrease in the reduced glutathione (GSH)/oxidized glutathione (GSSG) ratio and an upregulation of reactive oxygen species (ROS) levels, which impairs the formation and maintenance of memory CD8^+^ T cells [58]. After the activation of AMPK mediated by metformin (Met), the metabolic behavior of the cells shifted from glycolysis to fatty acid oxidation (FAO), which improved T cell survival and promoted differentiation of memory CD8^+^ T cells [59]. Following acute lymphocytic choriomeningitis virus infection in mice, a new bioenergetic bivalve-derived drug, IM156, was observed to enhance memory CD8^+^ T cell differentiation by affecting the mTOR pathway to attenuate glycolysis after activation of AMPK, which might be linked by a shift in metabolic modality to FAO [48]. Furthermore, it has been shown that changes in proteasome activity in CD8^+^ T cells regulate cellular metabolism by affecting *MYC* expression, a transcription factor that controls glycolysis and metabolic reprogramming, thus affecting their differentiation to effector and memory cells, in which high endogenous proteasome activity causes a bias towards differentiation to memory cells and low endogenous proteasome activity causes a bias towards differentiation to effector cells [60].

**Fig. 2:**
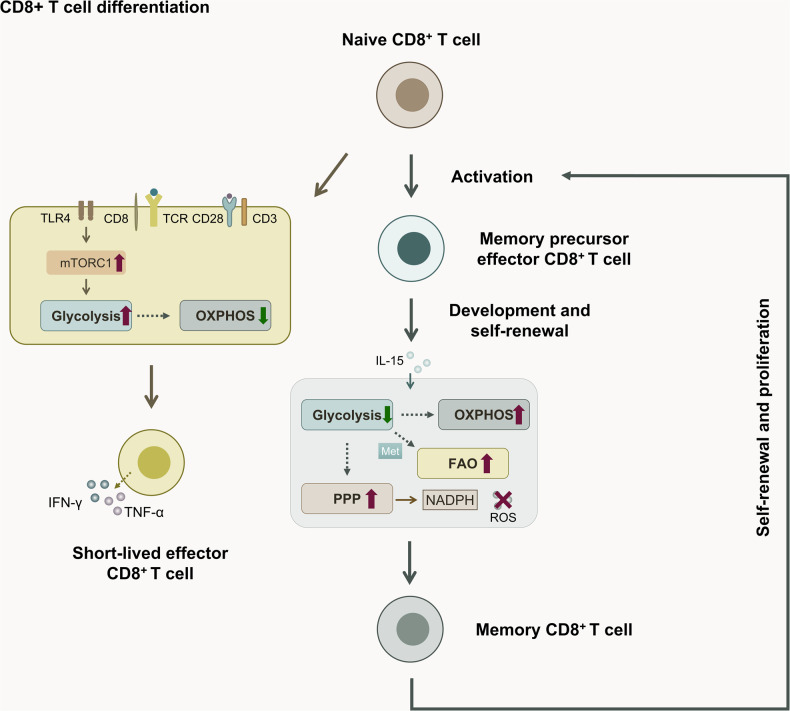
Effect of altered glycolysis levels on CD8^+^ T cell differentiation. In response to antigen stimulation, the metabolism of naive CD8^+^ T cells gradually shifts from OXPHOS to aerobic glycolysis to support further proliferation and differentiation into short-lived effector CD8^+^ T cells that secrete the effector factors IFN-γ and TNF-α. High glycolysis levels are not conducive to the differentiation of long-lived memory CD8^+^ T cells, and IL-15 is a key factor in the differentiation of CD8^+^ T cells by promoting glycoisomerization, and NADPH to scavenge intracellular free radicals, thereby maintaining the long-term survival of memory T cells. Met-mediated activation of AMPK promotes the conversion of cellular metabolism from glycolysis to fatty acid oxidation (FAO), thereby enhancing T cell survival and promoting the differentiation of memory CD8^+^ T cells. Long-lived memory CD8^+^ T cells reside in tissues and undergo renewed self-renewal and proliferation upon activation by the next stimulus.

The above studies suggest that the metabolic switch from OXPHOS to glycolysis in CD8^+^ T cells induces their functional differentiation towards effector phenotypes, while high glycolysis may severely impair the survival of long-lived memory CD8^+^ T cells (Fig. 2). It further suggests the possibility that we can activate CD8^+^ T cell differentiation toward effector cells and thus immune functions by targeting glycolysis; however, the underlying mechanisms and specific targets for this remain to be further investigated. More and more studies have focused on targeted inhibition of glycolysis to enhance its memory-type differentiation, and notably, the mTOR related signaling pathways play an important role in this process. Therefore, although it is feasible to control CD8^+^ T cell differentiation and further effector function by directly targeting glycolysis, there is a potential threat to the continued function of the immune system, and a reasonable metabolic switch is still the focus of ongoing research. Meanwhile, the metabolic shift from glycolysis to FAO may be linked to the differentiation of memory CD8^+^ T cells, but there is still insufficient research to elucidate the specific mechanism, exploring this aspect may provide new insights into the relationship between CD8^+^ T cell metabolism and differentiation, and also add new possibilities for CD8^+^ T cell immune response therapy.

## Effects of altered glycolysis levels on CD8^+^ T cell functions

### Effect of altered glycolysis levels on CD8^+^ T cell effector cell functions

The differentiation of CD8^+^ T cells from the naive to the effector state involves the upregulation of glucose-dependent metabolism, and glycolysis supplies energy to effector CD8^+^ T cells, which in turn exert their immune effector functions. CTLs are key players in the elimination of tumor or pathogen-infected cells via the phagocytic granule (LG) and Fas ligand (FasL) pathways. However, the consumption of glucose leads to severe damage to the cytotoxic function of CTLs. Strengthening glucose absorption to promote glycolysis does not affect CTLs proliferation, but greatly enhances their killing efficiency [61], indicating that changes in CD8^+^ T cell self-glycolysis levels are crucial for them to perform function of eliminating tumor or pathogen-infected cells.

Rapid production of IFN-γ following activation of effector CD8^+^ T cells through the TCR and costimulatory receptor CD28-mediated stimulation, or through cognitive interactions, is associated with increased glycolytic flux [62]. Furthermore, TCR transgenic can adjust the OT-I CD8^+^ T cell activation threshold, causing metabolic reprogramming of cells, with a marked increase in both glycolysis and OXPHOS, which in turn promotes autocrine IL-4 production [63]. Depriving glucose or inhibiting glycolysis with 2-DG can selectively inhibit the production of IFN-γ, but not IL-2 [64, 65]. In mouse CD8^+^ T cells, branched-chain amino acids (BCAA) accumulation increases cellular glycolysis and OXPHOS through FOXO1-dependent upregulation of GLUT1 level, thereby enhancing the anti-tumor immune function of CD8^+^ T cells., while BCAA supplementation also helps to improve the clinical efficacy of anti-PD-1 immunotherapy on tumors [66]. After activation of mouse CD8^+^ T cells using the pan-β-adrenergic receptor (AR) agonist isoprenaline (ISO), assays showed a decrease in their GLUT1 expression, and further studies revealed a significant decrease in glucose uptake and glycolysis compared with non-ISO activated CD8^+^ T cells, as well as impaired mitochondrial function, ultimately inhibiting CD8^+^ T cell activation and effector functions [67]. Meanwhile, in C57BL/6JJcl mice fed on a high-fat, high-sucrose diet (HFS), the glycolysis/basal respiration ratio was significantly reduced and the multifunctionality of CD8^+^ PD-1^+^ T cells was suppressed, whereas Met increased glycolysis and restored antigen-specific and non-specific cytokine production [68]. In addition, p110δ signaling affects CD8^+^ T cell activation, proliferation, and effector cytokine production by activating AKT and through regulating TCR-activated OXPHOS and aerobic glycolysis [41]. By contrast, TLR7 stimulates metabolic changes in CD8^+^ T cells through regulation of the AKT-mTOR pathway and the downstream transcription factor IRF4, including glucose uptake and glycolysis upregulation, thereby promoting the effector functions of CD8^+^ T cells in vitro. In contrast, corresponding deprivation of the mTOR pathway and IRF4 expression impairs T cell activation and function [53, 69]. Furthermore, Pellino E3 ubiquitin protein ligase 1 (Peli1) further regulates the mTORC1 repressor proteins TSC1 and TSC2 when stimulated by TCR signaling and growth factors, suggesting that Peli1 might affect CD8^+^ T cell glycolysis and thus regulate their antitumor immune function by mediating mTORC1 [70]. Furthermore, CD8^+^ T cells lacking the gene encoding autophagy related 5 (Atg5) acquired an effector phenotype, causing histone methylation, as well as upregulation of glycolytic and immune response genes, resulting in greater production of IFN-γ and TNF-α [71]. Upon FOXP3 overexpression, CD8^+^ T cells exhibit enhanced glucose and FA uptake and intracellular lipid accumulation. The enhancement of glycolysis, FA metabolism and OXPHOS activity helps to compensate for the loss of ATP production driven by mitochondrial respiration, improve their proliferative capacity and cytotoxicity, which in turn exerts anti-tumor effects [72].

Currently, studies have shown that targeted regulation of glycolysis may play an important role in the effector function of CD8^+^ T cells in various diseases. For instance, CD8^+^ T cells act on rheumatoid arthritis (RA) through releasing pro-inflammatory and cytolytic mediators. In RA, CD8^+^ T cells act through the release of pro-inflammatory and cytolytic mediators. CD8^+^ T cells in the blood of patients with RA meet their metabolic requirements through aerobic glycolysis, and inhibition of LDHA by using FX11 resulted in decreased adipogenesis, CD8^+^ T cell migration and proliferation, and CD8^+^ T cell effector function, increased ROS production, and loss of their ability to induce healthy B cells to develop a pro-inflammatory phenotype [73]. In addition, upregulation of FA transporter-binding protein 4 (FABP4) and G protein-coupled receptor 84 (GPR84) expression on the surface of effector and memory CD8^+^ T cells in RA patients concomitantly increases FA uptake, and further blocking FA metabolism in vitro would seriously impair the effector function of CD8^+^ T cells and further in vitro blockade of FA metabolism would severely impair the effector function of CD8^+^ T cells [74]. In melanoma, the highly acidified microenvironment induces G protein-coupled receptor (Ogr1) expression in T cells, while inhibiting Ogr1 reactivates CD8^+^ T cells and has cytotoxic effects by reducing the activity of high glycolytic, resulting in a relatively less acidified TME and tumor suppression [75]. Interestingly, in studies on lymph nodes (LNs), a low pH acidic environment inhibited T cell monocarboxylate transporters (MCTs), causing negative feedback regulation of glycolysis and further inhibiting the effector function of CD8^+^ T cells; however, this did not prevent the initial activation of nascent T cells by dendritic cells [76]. Furthermore, in obese mice with spontaneous onset of mammary tumors, ablation of T-cell STAT3, or treatment with FAO inhibitors, reduced FAO to increase glycolysis and CD8^+^ T effector cell function, thereby inhibiting mammary tumor development [77]. In clear cell renal cell carcinoma (ccRCC), ccRCC CD8 tumor infiltrating lymphocytes (TILs) cannot efficiently take up glucose or undergo glycolysis, and the mitochondria are small, fragmented, hyperpolarized, and produce large amounts of ROS, resulting in impaired cellular function and metabolism. In contrast, the activation of TILs can be partially restored, and their metabolic function improved by providing pyruvate to compensate for the defective glycolysis or using scavengers to neutralize mitochondrial ROS [78]. The use of bezafibrate facilitates the proliferation of nascent T cells and improves the effector function of CTLs by activating their mitochondria and upregulating OXPHOS and glycolysis [79].

Therefore, for effector CD8^+^ T cells, changes in their glycolysis play an important role in the production of IFN-γ, whereas downregulation of glycolysis levels is detrimental to their production of relevant cytokines and immune functions. Therefore, it is crucial to find ways to target glycolysis to restore CD8^+^ T cell effector functions. It is worth mentioning that, although the balance between glycolysis and OXPHOS play an important role in the CD8^+^ T cell differentiation and proliferation, glycolysis, FA metabolism and OXPHOS together provide energy for the effector function while glycolysis makes a difference in supporting their survival and development of effector CD8^+^ T cells. It suggests that there is an intricate link between the metabolism of CD8^+^ T cells and their differentiation and function. It is not enough to clarify the specific function of glycolysis, but it’s essential to investigate the relationship between various metabolic pathways and CD8^+^ T cells to provide theoretical basis for targeting their metabolism for therapy. In addition, numerous studies have shown that the impairment of CD8^+^ T cell effector function caused by glycolysis downregulation might be associated with both mitochondrial function and ROS production, thus providing a new direction to better restore adaptive immune function by targeting glycolysis while focusing on mitochondrial function; however, the specific mechanism of action remains to be investigated in depth.

### Effect of altered glycolysis levels on CD8^+^ T cells memory-type cell function

After antigen clearance, most effector CD8^+^ T cells (Teffs) undergo activation-induced cell death, and only a small fraction become long-lived memory CD8^+^ T cells [80]. For memory CD8^+^ T cells, glycolysis has different effects on their survival and functional performance at different times. Neonatal CD8^+^ T cells exhibited higher glycolytic activity than adult CD8^+^ T cells after infection, which might be caused by age-related differences in lin-28 homolog B (Lin28b) expression. Importantly, the impaired formation of neonatal memory CD8^+^ T cells could be restored when glycolysis was inhibited using drugs, suggesting that neonatal CD8^+^ T cells are inherently biased to undergo glycolytic metabolism after infection, which impairs their ability to develop into memory CD8^+^ T cells early in life [81]. The decrease of glycolysis and OXPHOS activity of CD8^+^ T cells caused by the deletion of nucleus accumbens-associated protein 1 (NAC1) contributes to the enhancement of memory formation of virus Ag-specific CD8^+^ T cells after virus infection [82]. Meanwhile, for stationary CD8^+^ T memory (Tm) cells, FAO is their predominant source of energy [83], indicating that highly active glycolysis is not critical for the differentiation and survival of CD8^+^ Tm cells. When encountering a homologous antigen for the second time, CD8^+^ Tm cells are reactivated, which is beneficial for preventing viral infection and malignant tumors [84]. Moreover, reactivated CD8^+^ Tm cells proliferate faster and in greater numbers than naive T cells [85, 86]. Increasingly, studies suggest that a rapid metabolic switch to glycolysis is essential during the reactivation process of CD8^+^ Tm cells [87], which also contradicts their resting metabolic mode. Meanwhile, CD8^+^ Tm cells use the gluconeogenic pathway to synthesize glycogen, which is abundant in Tm cells but not in naive or effector T cells [88]. These endogenous glycogen degradations provide a carbon source to meet immediate energy needs during the reactivation of CD8^+^ Tm cells [85].

During metabolism, mTORC2 rapidly activates AKT, which inhibits glycogen synthase 3β (GSK3β) at the mitochondrial-endoplasmic reticulum (ER) junction. The recruitment of hexokinase I (HK-I) to the voltage-dependent anion channel (VDAC) on mitochondria promotes respiration by promoting metabolite influx into mitochondria, which is required for the rapid production of IFN-γ in memory T cells [89]. Under hypoxic pressure culture conditions, upregulation of the levels of glycolytic pathway-related proteins such as GLUT1 and GLUT3 in memory CD8^+^ T cells lead to an increase in glycolysis level, thereby promoting their effector functions of IFN-γ and granzyme B production, but the ability of producing TNF-α and IL-2 is limited. Hypoxia conditions also cause the downregulation of OXPHOS and TCA related protein levels in memory CD8^+^ T cells, and conversely, hyperoxic conditions can promote activation of OXPHOS levels in naive and memory CD8^+^ T cells. Thus, reactivation of memory CD8^+^ T cells under hypoxia appears to impair cytokine production but enhances effector functions, including IFN-γ and granzyme B production [90]. In contrast, microRNA miR-143 was shown to increase glycolytic uptake and glycolytic activity through upregulation of GLUT1, promoting memory T cell differentiation and metabolic reprogramming, and further enhancing the antitumor effects of T cells [91]. In addition, within hours of systemic bacterial infection, acetate accumulates in serum, and upon uptake by memory CD8^+^ T cells, stress levels induced by acetate expand cellular acetyl coenzyme A via ATP citrate lyase and promote the acetylation of glyceraldehyde-3-phosphate dehydrogenase (GAPDH), which catalyzes glycolysis while increasing GAPDH activity, thereby facilitating the response of fast memory CD8^+^ T cells to exert superior immune control [92]. In the liver, assays revealed that memory CD8^+^ T cells generated by liver sinusoidal endothelial cells (LSECs) following TLR4 activation exhibited high levels of mitochondrial respiration and constitutively low levels of glycolysis to support their scavenger and sentinel functions [93].

**Fig. 3:**
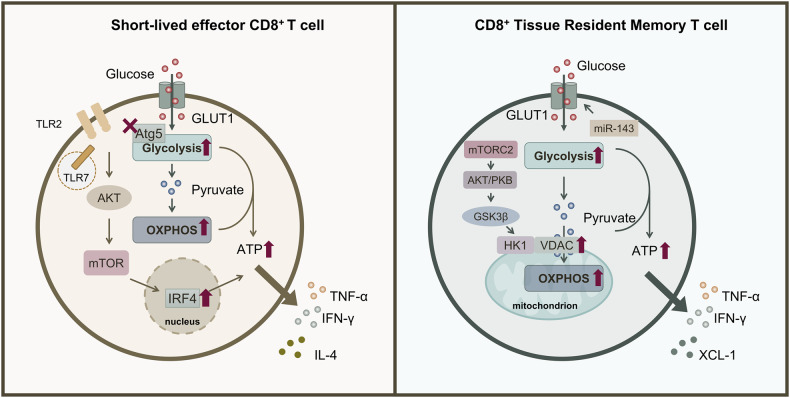
Effect of altered glycolysis levels on CD8^+^ T cell effector and memory cell functions. For short-lived CD8^+^ T cells, deletion of Atg5 would promote glycolytic activity and OXPHOS, which in turn would lead to energy production. Meanwhile, TLR2 and TLR7 stimulate upregulation of glucose uptake and glycolysis in CD8^+^ T cells through regulation of the AKT-mTOR pathway and the downstream transcription factor IRF4, thereby promoting effector functions including IL-4, IFN-γ, and TNF-α secretion in CD8^+^ T cells in vitro. For tissue-resident memory CD8^+^ T cells, during metabolism, mTORC2 promotes respiration by activating PKB or AKT to inhibit GSK3β at the mitochondrial-endoplasmic reticulum (ER) junction, leading to recruitment of HK-I to the voltage-dependent anion channel (VDAC) of mitochondria, thereby promoting metabolite influx into mitochondria to facilitate respiration and thus the rapid production of IFN-γ. Meanwhile, miR-143 targets *GLUT1* to promote glucose flux and glycolytic activity, further promoting memory-type functions.

Thus, while high glycolysis is detrimental to the development of early memory CD8^+^ T cells, a rapid transition from metabolism to glycolysis can facilitate their reactivation and performance of immune control functions about preventing viral infection and malignant tumors later in infection; however, for tissue-resident memory CD8^+^ T cells, high glycolysis levels are not conducive to their continued survival (Fig. 3). The mechanism by which glycolysis affects IFN-γ production by memory CD8^+^ T cells is clear and provides a reference for targeting glycolysis to further enhance memory CD8^+^ T cell function; however, the detrimental effect of high glycolysis on the long-term residence of memory CD8^+^ T cells will increase the difficulty of affecting CD8^+^ T memory function by targeting glycolysis. In contrast to naive CD8^+^ T cells, reactivated CD8^+^ Tm cells tend to proliferate faster and have a larger number, so the awakening of their functions deserves attention, and it can be argued that glycolysis plays a crucial role in this process. There is still a big gap in the research on the relationship between glycolysis and OXPHOS, TCA cycle and FAO during the reactivation and functional exertion of memory CD8^+^ T cells.

### Effect of glycolysis on CD8^+^ T cell dysfunction and recovery

The functional integrity of CD8^+^ T cells is closely linked to metabolic reprogramming, and metabolic imbalance of glycolysis and OXPHOS will cause dysfunction of CD8^+^ T cells, which can be ameliorated by further glycolytic restoration, it also provides new ideas for restoring the function of CD8^+^ T cells. SUMO specific peptidase 7 (SENP7) deficient CD8^+^ T cells exhibited reduced glycolysis and OXPHOS, leading to diminished proliferation in vitro and reduced antitumor function in vivo. SENP7 acts as an oxidative stress sensor that maintains the metabolic capacity and effector functions of CD8^+^ T cells [94]. Moreover, ROS production by CD8^+^ T cells triggers cell membrane SENP7-mediated de-SUMOylation of phosphatase and tensin homolog (PTEN), thereby promoting PTEN degradation and preventing PTEN-dependent metabolic defects. In contrast, ROS in T cells restrict the cell membrane translocation of SENP7 and inhibit the metabolic and functional activity of CD8^+^ T cells in human colorectal cancer samples [94]. TILs found in human and murine CD8^+^ melanomas are metabolically impaired, with defects in both glycolysis and oxidative metabolism. This was shown to be caused by downregulation of the activity of Enolase 1, a key enzyme in the glycolytic pathway, which inhibits the glycolytic activity of CD8^+^ TILs. Provision of pyruvate, a downstream product of Enolase 1, could bypass this inactivity and promote glycolysis and OXPHOS, thereby improving the effector function of CD8^+^ TILs [95]. The transcription factor nuclear factor of activated T cells 1 (NFATC1) controls the cytotoxicity of murine CTLs and the production of associated cytokines by affecting the expression of the glycolysis-related gene *Hk2*, which shifts the metabolic mode of CD8^+^ T cell metabolism from OXPHOS to a metabolic switch for glycolysis, and IL-2 restores this change [96]. Interestingly, in studies on influenza A virus (IAV), it was found that CD4^+^ T cells contribute to CTL formation and establish memory, and that CD4^+^ T cells contribute to the recall of memory CD8^+^ T cells by promoting molecular pathways that enhance CTLs’ respiratory capacity and their ability to participate in glycolysis upon reactivation, which would prevent their failure [97].

T cell senescence is thought to contribute to the decline in immune function; however, the pathways mediating senescence in these cells are not well understood, and studies suggest that CD8^+^ T cell failure might be associated with their glycolytic activity. After evaluation of T cell populations from healthy volunteers, human CD8^+^ effector memory T cells expressing the naive T cell marker CD45RA (also known as protein tyrosine phosphatase receptor type C) were found to have many features of cellular senescence, in which the senescent CD45RA-expressing population was involved in anaerobic glycolysis to generate energy for effector functions [98]. Meanwhile, suppressing the presence of p38 mitogen-activated protein kinase (MAPK) signaling in senescent CD8^+^ T cells increased their proliferation, telomerase activity, mitochondrial biogenesis, and somatic energy, and enhanced the interaction between p38 interacting protein (p38IP) and autophagy protein 9 (ATG9), and blocking p38 MAPK signaling independent of the mTOR pathway would reverse their failure [98]. Furthermore, acylglycerol kinase (AGK) activity mediates the activation of PI3K-mTOR signaling, causing elevated CD8^+^ T cell glycolysis, which is required to maintain CD8^+^ T cell metabolism and functional robustness [99].

By contrast, the tumor suppressor myostatin works by limiting the activity of mTORC1 and thus inhibits its glycolysis-mediated activity to maintain T-cell function and prevent dysfunction as a result of their exhaustion [100]. This is due to the increasing number of studies showing that although high glycolysis level is essential for maintaining the effector functions of CD8^+^ T cells, it also helps to restore their functional changes caused by metabolic dysregulation. However, an over-reliance on glycolysis will cause deterioration of mitochondrial metabolism in CD8^+^ T cells, leading to metabolic arrest and functional decline. Following chronic *Mycobacterium tuberculosis* (Mtb) infection, the mitochondrial metabolism of CD8^+^ T cells deteriorates, leading to an increased dependence on glycolysis and enhanced production of inflammatory cytokines; however, over time, cellular metabolism becomes quiescent and their function declines. In contrast, Met can be reactivated, favoring the generation of Mtb-specific CD8^+^ T cell populations with autonomously improved metabolism [101]. In addition, CD8^+^ T cell failure in human immunodeficiency virus (HIV) infection was found to be characterized by reduced glycolytic activity, enhanced OXPHOS requirement, mTOR dysregulation, and reduced cytoplasmic GAPDH [102]. HIV-specific CD8^+^ T cells from spontaneous controllers are largely dependent on glucose, whereas cells from HIV controllers (HICs) have more diverse metabolic resources, which enhances both their survival potential and their ability to develop anti-HIV effector functions. In contrast, after IL-15 treatment, HIV-specific CD8^+^ T fine cells from non-controllers undergo metabolic reprogramming in vitro and are less glucose-dependent, which enhances the antiviral capacity of HIV-specific CD8^+^ T cells from non-controllers [103]. Meanwhile, GLUT1 (hi) Hepatitis B virus (HBV)-specific T cells are dependent on the glucose supply, unlike the more functional cytomegalovirus (CMV)-specific T cells, which can rely on OXPHOS in the absence of glucose. The inability of HBV-specific T cells to shift their metabolism from glycolysis to OXPHOS causes an increase in mitochondrial size and a decrease in mitochondrial potential, resulting in mitochondrial dysfunction. In contrast, IL-12, which restores the effector function of HBV-specific T cells, reduces their dependence on glycolysis by increasing the mitochondrial potential [104]. Currently, relevant drugs prevent the dysfunction of CD8^+^ T cells by targeting mTOR activity directly. An emerging therapeutic bile acid, 4-norursodeoxycholic acid (NorUDCA), is used in primary sclerosing cholangitis (PSC) to target mTORC1 signaling to affect glycolysis and prevent CD8^+^ T cells from undergoing further energy damage, thus directing them back on the right path to function properly to defend against viruses and cancer cells [105]. In colorectal cancer (CRC) cells, the triterpenoid TER of *Flavobacterium vulgare* mediated glycolytic activity by limiting mTOR activity, increased the expression of multiple metabolic receptors, promoted CD8^+^ T cell metabolism and activation, while preventing CD8^+^ T cell dysfunction, reversed the effector dysfunction of CD8^+^ T cells, secreted more IFN-γ, IL-10, TNF-α, and TGF-β, thereby contributing to enhanced T cell recognition [106].

In addition, PD-1 signal plays a key role in regulating the balance between mTOR-dependent anabolic glycolysis and FAO programs to meet the bioenergetic demands of quiescent memory CD8^+^ T cells. Furthermore, PD-1 signaling plays a key role in regulating the balance between mTOR-dependent anabolic glycolysis and FAO programs to meet the bioenergetic demands of quiescent memory CD8^+^ T cells. In contrast, upregulation of glycolysis and impairment of fatty acid metabolism caused by PD-1 deficiency would favor memory CD8^+^ T cell survival [107]. This suggests that highly active glycolysis is detrimental to memory CD8^+^ T cell differentiation and the survival of long-lived memory CD8^+^ T cells. In contrast, binding of the programmed death-1 (PD-1) receptor to its ligand (PD-L1/2) conveys inhibitory signals that promote the exhaustion of activated T cells, and blockade of the PD-1 pathway is widely used in cancer therapy. RNA sequencing evaluation revealed that PD-1 primarily targets the glycolytic and OXPHOS pathways, causing severe functional and structural changes in cellular mitochondria to efficiently utilize FAO, leading to the exhaustion of activated T cells, as well as reduced IFN-γ production following T cell activation by anti-CD3 and CD28 activating antibodies [108]. CD8^+^ T cells from patients treated using long-term antiretroviral therapy (LT-ART) showed increased frequency of T cell immunoglobulin mucin 3 (TIM3) ^+^ programmed cell death 1 PD1^-^ cells, and treatment with combined anti-PD1 and anti-T cell immunoreceptor with Ig and ITIM domains (TIGIT) antibodies plus glycolysis-promoting drugs restored dysfunction in CD8^+^ T cells after short-term antiretroviral therapy (ST-ART) [109].

**Fig. 4:**
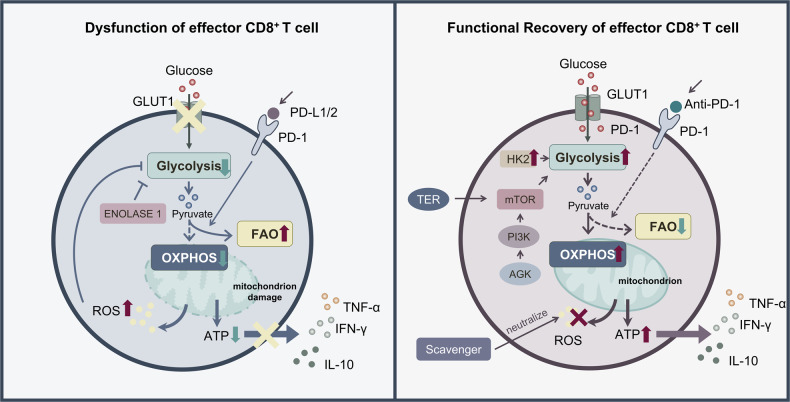
The effect of altered glycolysis levels on CD8^+^ T cell dysfunction and recovery. Dysfunction of CD8^+^ T cells often occurs in the immunosuppressive tumor microenvironment when the binding of PD-1 to PD-L1/2 transmits inhibitory signals that reduce the entry of pyruvate into OXPHOS and instead into fatty acid oxidation, while causing mitochondrial damage and hyperpolarization to produce large amounts of ROS. This inhibits glycolytic activity and affects cellular metabolism and energy production, ultimately contributing to the dysfunction of activated CD8^+^ T cells and affecting the secretion of cytokines associated with their effector functions. In the face of dysregulated CD8^+^ T cells, combined treatment with anti-PD-1 and glycolysis-promoting drugs can partially restore their function. The use of anti-PD-1 drugs can inhibit the entry of pyruvate in large amounts into fatty acid oxidation, promoting OXPHOS and energy production. Meanwhile, AGK kinase activity mediates PI3K-targeted mTOR activation, causing elevated glycolysis, while the presence of transcription factor NFDTC1 regulates HK2 expression and promotes glycolysis. In addition, the use of ROS eliminators neutralizes ROS, eventually restoring CD8^+^ T cell function and the further secretion of IL-10, IFN-γ, and TNF-α.

Thus, the imbalance of glycolysis and OXPHOS often leads to dysfunction of CD8^+^ T cells, and glycolysis might affect the partial failure of CD8^+^ T cells through the mTOR pathway and adversely affect the production of IFN-γ. Reactivation of glycolysis will help to preventing its failure and restoring the resulting dysfunction. However, excessive glycolysis dependence may often lead to metabolic arrest and consequent functional decline of CD8^+^ T cells (Fig. 4). It’s crole that glycolysis plays in the function of CD8^+^ T cells, and therefore there is still significant uncertainty about the application of targeting glycolysis to regulate CD8^+^ T cells function. In addition, it is worth noting that, in addition to compensating the glycolytic defect to restore CD8^+^ T cell function, how to deal with the mitochondrial dysfunction and ROS production caused by metabolic dysregulation will also be important to improve CD8^+^T cells function. In addition, the balance between glycolysis and FAO is associated with the long-term survival of memory CD8^+^ T cells, and there are still insufficient studies on the mechanisms involved. Meanwhile, an increasing number of studies are now finding that PD-1 signal affects the balance of glycolysis and OXPHOS by mediating mTOR in CD8^+^ T cell, in turn delivering inhibitory signals, which also provides new ideas for combined anti-PD1 and targeted CD8^+^ T cell glycolytic activity therapy.

## Challenges and prospects

According to current studies, glycolysis provides energy for the further proliferation the of CD8^+^ T cells after activation, and the appropriate regulation of glycolysis level of CD8^+^ T cells also helps to promote their timely death to prevent excessive proliferation. Of course, the proliferation of CD8^+^ T cells does not completely depend on glycolysis, as high level of OXPHOS also plays an important role. In addition, the metabolic transition from OXPHOS to glycolysis in CD8^+^ T cells facilitates their differentiation to effector CD8^+^ T cells and the secretion of cytokines, such as IFN-γ, to exert their immune functions, which provides the basis to identify and target the regulation of relevant genes to exert immunotherapy. However, it’s often detrimental to the survival of long-lived memory cells. For memory CD8^+^ T cell differentiation, a metabolic switch from glycolysis to FAO may play an important role. After differentiation, glycolysis, FA metabolism, and OXPHOS work together to provide the energy for effector CD8^+^ T cells to produce relevant cytokines and perform immune functions. Although high glycolytic activity is detrimental to the differentiation and development of early memory CD8^+^ T cells, it is essential for the reactivation of CD8^+^ Tm cells, and the function of glycolysis in reactivation seems to be relative to that of OXPHOS and TCA. Although there are in-depth studies on how glycolysis affects the function of effector CD8^+^ T cells in the short term, there are fewer studies on how glycolysis affects the pre-development and late survival and transformation of memory CD8^+^ T cells, which is not conducive to further targeted regulation of the long-term function of CD8^+^ T cells. As the disease progresses, imbalances in glycolysis and OXPHOS often leads to dysfunction of CD8^+^ T cells, and the downregulation of glycolytic activity often results in CD8^+^ T cell failure, selectively increasing glycolysis will further restore these functions. Notably, it has also been suggested that excessive glycolytic dependence may lead to metabolic quiescence and consequent functional decline of CD8^+^ T cells, so reducing the dependence of CD8^+^ T cells on glycolysis will help prevent their dysfunction and maintain their immune functions. It can be said that from the activation of naive CD8^+^ T cells, glycolysis plays an important role as a functional machine involved in their subsequent proliferation and differentiation, while for CD8^+^ T cells at different periods, glycolysis has different effects on their survival and function, therapies that target key aspects of glycolysis still need to be selected according to the specific situation. However, current therapeutic modalities often exert short-lived effector functions but neglect long-lived CD8^+^ T cell survival and residency, to the detriment of their reactivation. In addition, studies have shown that the activation and functional changes of CD8^+^ T cells caused by glycolytic changes are often accompanied by mitochondrial dysfunction and changes in other metabolic pathways, such as OXPHOS, fatty acid synthesis, and amino acid metabolism. Therefore, focusing on the overall metabolic level of CD8^+^ T cells and metabolism-related organelle functions might be the way forward to achieve immunotherapeutic effects.

At the molecular level, relevant signal pathways including the key molecule mTOR play an important role in the above process, which provides direction for targeting CD8^+^ T cells glycolysis to regulate their immune function. Meanwhile, PD-1 delivers inhibitory signals by mediating mTOR to affect the metabolic balance of CD8^+^ T cells glycolysis and OXPHOS, which also provides a new insight for combined anti-PD-1 and targeting CD8^+^ T cells glycolytic activity therapy. Hypoxia-inducible factor-1α (HIF-1α) induces a shift in metabolic pathways to glycolysis by forming a dimer with HIF-1β and binding to intranuclear hypoxia response element (HRE) target genes to induce the expression of GLUT1 and other enzymes in glycolysis [110], playing an important regulatory function in the survival and function of tumor cells and other immune cells [111]. And the enhanced HIF-1α-dependent growth of tumors is associated with increased multifunctionality of CD8^+^ TILs [112]. In addition, HIF-1α upregulation contributes to the metabolic switch from FAO to glycolysis, thereby promoting effector T cell differentiation [113, 114]. For CD4^+^ T cells, HIF-1α-mediated glycolysis activity and metabolic reprogramming are important for their differentiation and function [115–118]. At present, studies on the molecular mechanisms of glycolysis changes in CD8^+^ T cells have only addressed the PI3K/AKT/mTOR pathway, with less mention of, the key factor HIF-1 [110], suggesting that, besides the transcription factor IRF4 and the key molecule mTOR, there are other key molecules involved in image glycolysis and thus regulate CD8^+^ T differentiation and function.

However, there are still many gaps in the existing studies, such as the metabolic shift from glycolysis to FAO to support memory CD8^+^ T cell differentiation [48], and the specific mechanisms underlying this remain unclear. For effector CD8^+^ T cells, although glycolysis is important for their effector functions, glycolysis, FA metabolism and OXPHOS often work together, and the linkages between metabolism within CD8^+^ T cells is still indistinct. Therefore, so it is crucial to investigate the links between various metabolic pathways and CD8^+^ T cells to provide a theoretical basis for targeting their metabolism. Moreover, there are still vacancies in the study of the relationship between glycolysis and OXPHOS, the TCA cycle and FAO during the reactivation and function of memory CD8^+^ T cells. Furthermore, it is worth considering why glycolysis and OXPHOS play a synergistic role in the function of effector CD8^+^ T cells, while reactivated CD8^+^ Tm cells often exhibit a high level of glycolysis and OXPHOS and FA metabolism are downregulated? Can glycolysis itself support their activation and function? Or other metabolic pathways may be required for auxiliary functions? At the same time, high *HK2* expression in lung cancer tissues is associated with a reduced ratio of CD8^+^ T cells to Tregs [119], while lactate accumulation induced by *LDHA* transcript levels in melanoma cells induces CTL failure and apoptosis by inhibiting cytokine production, while impairing their cell killing capacity [120]. In mouse colon cancer cells MC38, lactate can increase the stemness of CD8^+^ T cells, and also increase the expression of TCF-1 in human CD8^+^ T cells in vitro, while reducing their apoptosis [121]. In addition, the metabolic competition between tumor cells and T cells tends to affect the anti-tumor response of CD8^+^ TILs [122]. Thus, in addition to the glycolysis of CD8^+^ T cells themselves, changes in the glycolysis of their target cells, such as tumor cells may affect the survival and function of CD8^+^ T cells by altering the microenvironment. So, will the changes of glycolysis process of other immune cells like CD4^+^ T cells and macrophages in the TME affect the proliferation, differentiation, and function of CD8^+^ T cells? Are there other metabolites produced by other cells in the microenvironment, such as D-2HG [123], that are taken up by CD8^+^ T cells, affecting their glycolysis and thus changing their differentiation and function? At present, there are few studies on effects between various immune cells. It’s necessary to further clarify the potential metabolism-mediated links between immune cells in the TME to provide viable options for immunotherapy through modulation of CD8^+^ T cell activation and function.

## Availability of data and materials

Not applicable.
